# Emerging Infectious Disease Implications of Invasive Mammalian Species: The Greater White-Toothed Shrew (*Crocidura russula*) Is Associated With a Novel Serovar of Pathogenic *Leptospira* in Ireland

**DOI:** 10.1371/journal.pntd.0005174

**Published:** 2016-12-09

**Authors:** Jarlath E. Nally, Zbigniew Arent, Darrell O. Bayles, Richard L. Hornsby, Colm Gilmore, Siobhan Regan, Allan D. McDevitt, Jon Yearsley, Séamus Fanning, Barry J. McMahon

**Affiliations:** 1 Infectious Bacterial Diseases Research Unit, National Animal Disease Center, Agricultural Research Service, United States Department of Agriculture, Ames, Iowa, United States of America; 2 University Centre of Veterinary Medicine JU-UAK, University of Agriculture, Krakow, Poland; 3 OIE Leptospirosis Reference Laboratory, Veterinary Sciences Division, AFBI, Belfast, Northern Ireland, United Kingdom; 4 UCD School of Agriculture & Food Science, University College Dublin, Belfield, Dublin, Ireland; 5 UCD School of Biology & Environmental Science and UCD Earth Institute, University College Dublin, Belfield, Dublin, Ireland; 6 UCD Centre for Food Safety, School of Public Health, Physiotherapy & Sports Science, University College Dublin, Belfield, Dublin, Ireland; 7 Ecosystems and Environment Research Centre, School of Environment and Life Sciences, University of Salford, Salford, United Kingdom; Instituto Butantan, BRAZIL

## Abstract

The greater white-toothed shrew (*Crocidura russula*) is an invasive mammalian species that was first recorded in Ireland in 2007. It currently occupies an area of approximately 7,600 km^2^ on the island. *C*. *russula* is normally distributed in Northern Africa and Western Europe, and was previously absent from the British Isles. Whilst invasive species can have dramatic and rapid impacts on faunal and floral communities, they may also be carriers of pathogens facilitating disease transmission in potentially naive populations. Pathogenic leptospires are endemic in Ireland and a significant cause of human and animal disease. From 18 trapped *C*. *russula*, 3 isolates of *Leptospira* were cultured. However, typing of these isolates by standard serological reference methods was negative, and suggested an, as yet, unidentified serovar. Sequence analysis of 16S ribosomal RNA and *secY* indicated that these novel isolates belong to *Leptospira alstonii*, a unique pathogenic species of which only 7 isolates have been described to date. Earlier isolations were limited geographically to China, Japan and Malaysia, and this leptospiral species had not previously been cultured from mammals. Restriction enzyme analysis (REA) further confirms the novelty of these strains since no similar patterns were observed with a reference database of leptospires. As with other pathogenic *Leptospira* species, these isolates contain *lipL32* and do not grow in the presence of 8-azagunaine; however no evidence of disease was apparent after experimental infection of hamsters. These isolates are genetically related to *L*. *alstonii* but have a novel REA pattern; they represent a new serovar which we designate as serovar Room22. This study demonstrates that invasive mammalian species act as bridge vectors of novel zoonotic pathogens such as *Leptospira*.

## Author Summary

Leptospirosis is a global zoonotic disease. Pathogenic species of *Leptospira* are excreted in urine from asymptomatic carrier hosts which facilitates disease transmission to new hosts. To date, there are 10 species of pathogenic leptospires which comprise more than 200 serovars. Disease transmission of these strains is maintained by a wide range of domestic and wild animal species. In this work, we discovered that an invasive mammalian species, the greater white toothed shrew, which was first identified in Ireland in 2007, acts as a carrier for a species of leptospires never before identified in Ireland. Results demonstrate that invasive mammalian species act as bridge vectors of novel zoonotic pathogens such as *Leptospira*.

## Introduction

The greater white-toothed shrew (*Crocidura russula*) is an exotic species to Ireland first recorded in 2007[1], and now classified as an invasive mammalian species[2]. According to recent studies, this species is rapidly spreading with radial expansion estimates of approximately 5.5 km/yr[2]. The source of this invasive population is from Europe as opposed to North Africa[3], and evidence suggests that the greater white-toothed shrew is associated with the local extinction of indigenous populations of the pygmy shrew (*Sorex minutus*)[2]. However, a comprehensive investigation on the One Health implications of this invasive species has yet to be performed.

Pathogenic species of *Leptospira* cause leptospirosis, a bacterial zoonotic disease with a global distribution affecting over one million people annually[4, 5]. Leptospires colonize the renal tubules of reservoir hosts, from where they are excreted via urine into the environment and survive in suitable moist conditions. Contact with infected urine, or contaminated water sources can result in disease since pathogenic leptospires can penetrate breaches of the skin, or mucosal surfaces, and disseminate haematogenously to cause a range of clinical symptoms from mild fever, to icteric Weil’s disease and pulmonary hemorrhage syndrome. In developed countries, leptospirosis is primarily a recreational disease, or occupational disease of farm workers, veterinarians, and slaughter plant workers. In developing countries, it is a socioeconomic disease perpetuated by rapid urbanization, rodent infestation and transmission via contaminated water sources associated with limited infrastructures and severe weather events. Both rodents and domestic farm animal species can serve as reservoir hosts of infection and sources of disease transmission to humans.

Leptospirosis is endemic in Ireland[6–12]. The mean annual incidence for 2009 was 5.6 *per* million inhabitants per annum, compared to that of 1.4 *per* million across the EU[13]. The predominant serovars associated with human infection were serovars Icterohaemorrhagiae and Hardjo, indicative of rodent/recreational and occupational exposure respectively. Rats are reservoir hosts for serovar Icterohaemorrhagiae whilst cattle act as reservoir hosts for serovar Hardjo[14]. Over 80% of Irish beef suckler herds, and more than 40% of individual beef producing animals, show evidence of exposure to leptospires[15]. Similarly, 79% of unvaccinated dairy herds were positive for antibodies to *Leptospira* by bulk tank milk testing[16]. Leptospirosis continues to be a leading cause of bovine abortion[17]. Other domestic animals species that show evidence of exposure to pathogenic leptospires in Ireland include pigs, sheep, horses and dogs[18–26].

There is clear evidence that invasive species act as vectors for pathogens and parasites that can have environmental conservation, and human health, implications. Globalization has facilitated the movement of exotic and invasive species, and a range of associated pathogens e.g. mosquitoes and West Nile Virus[27]. The combination of invasive species and degradation of ecosystems presents a substantial threat in relation to emerging infectious diseases[27, 28]. Novel pathogens can have devastating effects on naive communities; examples include the invasive grey squirrel (*Sciurus carolinensis*) which carries squirrelpox virus that severely adversely affected native red squirrels (*Sciurus vulgaris*) in Britain and Ireland[29, 30]; the introduced raccoon dog (*Nyctereutes procyonoides*) in Europe, which has an expanding range, and which can facilitate the spread of infectious diseases including echinococcosis, trichinellosis and rabies[31]. In this study, we identified that a recently introduced mammalian species (*C*. *russula*) in Ireland is a reservoir host for a novel strain of pathogenic *Leptospira*.

## Materials & Methods

### Greater white-toothed shrews

Greater white-toothed shrews (GWTS) were live-trapped and euthanized by cervical dislocation. All animal experimental procedures were performed in accordance with relevant guidelines and regulations, and as approved by the National Parks and Wildlife Service (NPWS) in Ireland and the Animal Research Ethics Committee in University College Dublin (AREC-13-24).

### Cultures

Kidneys were removed from GWTS at time of euthanasia and immediately processed for the culture of leptospires[32]. In brief, a single kidney was aseptically removed using a disposable forceps and scalpel and placed in 5 ml 1% Bovine Serum Albumin (BSA). The kidney was subsequently macerated with scalpels and the resulting mixture homogenized by passing it through a 10ml syringe (without needle attachment). Each tissue homogenate was serially diluted 10-fold (to a final dilution of 10^−3^) into 1% BSA and 500μl of this mixture was used to inoculate the surface of 10ml EMJH medium containing 200μg 5-Fluoruracil and 0.2% noble agar. Cultures were transported back to the laboratory and maintained at 29°C. Cultures were examined at weekly intervals by dark-field microscopy.

*L*. *alstonii* Serogroup Ranarum Serovar Pingchang Strain 80–412 and *L*. *alstonii* Serogroup Undesignated Serovar Sichuan Strain 79601 were sourced from the WHO/OIE Leptospirosis Reference Laboratory at the Royal Tropical Institute, The Netherlands. *L*. *alstonii* strains MS267, MS311 and MS316 were kindly provided by Department of Bacteriology, Faculty of Medical Sciences, Kyushu University, Japan.

Growth assessment in the presence of 8-azaguanine was performed as previously described[33]; in brief, leptospires were cultured in EMJH medium with 1% rabbit serum and 225 μg/ml 8-Azaguanine (A5284 8-Azaguanine, Sigma, St. Louis, MO). Duplicate tubes were inoculated with the shrew isolates while *Leptospira* biflexa (ATCC^®^ 23582^™^) was used as a positive control. Cultures were incubated at 30°C for 14 days. The cultures were counted by dark-field microscopy at days 1, 3, 5, 7 and 14 using a Cellometer^®^ disposable cell counting chamber (Nexcelom Bioscience).

### Serological typing of isolates

Serological strain identification was initially attempted by cross-agglutination. In this procedure, the Microscopic Agglutination Test (MAT) was carried out using a panel of 19 reference antisera against the 17 major pathogenic *Leptospira* serogroups[34–36]. The *Leptospira* serogroups tested included Australis (serovars Australis and Bratislava), Autumnalis, Ballum, Canicola, Celledoni, Cynopteri, Grippotyphosa, Hebdomadis, Icterohaemorrhagiae, Javanica, Louisiana, Mini, Pomona (serovar Pomona and Altodouro), Pyrogenes, Sejroe, Semaranga and Tarassovi. In addition, rabbit sera generated against each of the three shrew isolates were then tested against the panel of *Leptospira* antigens from the 17 serogroups mentioned above, and additionally against a panel of 9 antigens from serogroups comprised of: Andamana, Semaranga, Hursbridge, Sarmin, Lyme, Louisiana, Shermani (serovar Shermani and Aquaruna), Bataviae, Ranarum, and against one undesignated serogroup (serovar Sichuan).

### Restriction enzyme analysis

Four hundred ml culture grown from each shrew isolate of *Leptospira* was harvested and whole cell leptospiral DNA purified as previously described[18]. DNA concentration was estimated after spectrophotometric measurement using a Nanophotometer Pearl (Implen). Restriction endonuclease digestion with *EcoR*I, electrophoresis and gel analysis were carried out as previously described[18].

### Generation of antiserum

Rabbit sera were prepared as previously described with slight modification[34] and as licensed under the Animals (Scientific Procedures) Act (1986). In brief, rabbits were injected intraperitoneally at weekly intervals with live leptospires at a density of 2 x 10^8^ per ml. The weekly injected doses were 5, 10, 15, and 20 ml respectively. Rabbits were bled by cardiac puncture one week after the last injection.

### Genome sequencing

Genome sequencing was performed by the Centre for Genomic Research at the University of Liverpool. Genomic DNA material was purified with 1x cleaned Ampure beads (Agencourt) and the quantity and quality was assessed by Nanodrop and the Qubit assay. In addition, the Fragment Analyser (using a high sensitivity genomic kit) was used to determine the average size of the DNA and the extent of degradation. This procedure was also used at the steps indicated below to determine average fragment size of the DNA. DNA was sheared using Covaris G tubes by centrifugation at 7,000 rpm in an Eppendorf 5415R centrifuge. The fragment size was checked as before. DNA was purified with 0.5x ampure beads and treated with Exonuclease VII at 37°C for 15 minutes. The ends of the DNA were repaired as described by Pacific Biosciences protocol. Each sample was incubated for 20 minutes at 37°C with DNA Damage Repair Mix supplied in the SMRTbell library kit (Pac Bio). This was followed by 5 minutes incubation at 25°C with End Repair Mix. DNA was cleaned using 0.5x ampure and 70% ethanol washes. DNA was ligated to adapter sequences overnight at 25°C. Ligation was terminated by incubation at 65°C for 10 minutes followed by exonuclease treatment for 1 hour at 37°C. The SMRTbell library was purified with 0.5x ampure beads. The quantity of library and therefore the recovery was determined by Qubit assay and the average fragment size determined by Fragment Analyser. SMRTbell library was annealed to sequencing primer at values predetermined by the Binding Calculator (Pac Bio) and a complex made with the DNA Polymerase (P6/C4 chemistry). The complex was bound to Magbeads and this was used to set up 3 SMRT cells for sequencing. Sequencing was done using 240 minute movie times.

### Phylogeny

The 16S rRNA gene sequence identified within the newly sequenced organism described herein was used to retrieve 108 similar sequences from the Ribosomal Database Project (RDP) via the SeqMatch tool[37]. Sequences were aligned with MUSCLE[38], and divergent and ambiguously aligned alignment blocks were removed with Gblocks[39]. The modelTest feature of Phangorn[40] was used to calculate the Bayesian Information Criterion (BIC) for a variety of models, and guided the selection of the HKY model. The model parameters for computing the maximum likelihood of phylogeny were optimized using optim.pml, and bootstrap.pml was used to perform a bootstrap analysis[40]. The phylogenetic reconstruction with bootstrapped values assigned to the edges was graphically rendered with TreeDyn[41].

The *secY* gene sequence identified within the newly sequenced organism described herein was compared with other sequences of *secY* from the genus *Leptospira*, as retrieved from GenBank[42]. Sequences of *secY* were aligned with CLUSTAL W[43]. Phylogenic analysis was conducted with MEGA4[44] and the maximum likelihoods method was used for estimation of distance of aligned sequences[45].

### Experimental infection of hamsters

Golden Syrian hamsters were inoculated by intraperitoneal (IP) injection as previously described[46]. Groups of three hamsters each received 10^7^ of GWTS isolate #1, #2 or #3 IP respectively. Three hamsters acted as negative controls and received media alone. All animal experimental procedures were performed in accordance with relevant guidelines and regulations, and as approved by USDA Institutional guidelines.

### Microscopic agglutination test

The microscopic agglutination test was performed as previously described according to OIE guidelines[47].

### Fluorescent antibody test

The fluorescent antibody test was performed as previously described[32].

### Accession numbers

The annotated assembly for *L*. *alstonii* serovar Room22 strain GWTS#1 is available in GenBank under the accession numbers CP015217 (Chromosome I) and CP015218 (Chromosome II).

## Results

### Culture and serological classification of GWTS isolates of leptospires

Culture of leptospires was attempted from a single kidney in each of 18 trapped GWTS. Kidneys from three of the GWTS were culture positive as confirmed by dark-field microscopy and the isolates were named GWTS Isolate #1, #2 and #3 respectively.

Each GWTS isolate of *Leptospira* was tested against a standard panel of reference antisera, representing 19 serovars from 17 serogroups and representative of the geographical locale, for typing purposes, Table 1. No significant reactivity was detected between any GWTS isolate and any reference sera. In a further attempt to type each GWTS isolate, rabbit antisera specific for each GWTS isolate was then prepared and tested against an additional panel of reference strains of *Leptospira*, representing 9 serogroups, one undesignated serogroup, and 13 serovars, Table 2. Slight reactivity was detected by antisera specific for GWTS isolate #1 & #2 against serovar Shermani, which belongs to *Leptospira santarosai*. However, the lack of a consistently high MAT titre detected between GWTS isolate-specific antisera and reference antigen indicated an inconclusive serological typing classification of any of the GWTS isolates, and suggesting that they were of an as yet unidentified serovar.

**Table 1:** MAT titres of GWTS Isolates 1, 2 & 3 with reference antisera.

Reference antisera		Antigen		
Serogroup	serovar	GWTS-1	GWTS-2	GWTS-3
Australis	Australis (Ballico)	0	0	0
Australis	Bratislava	0	0	0
Autumnalis	Autumnalis	0	0	0
Ballum	Ballum	0	0	0
Canicola	Canicola	0	0	0
Celledoni	Celledoni	0	0	0
Cynopteri	Cynopteri	0	0	0
Grippotyphosa	Grippotyphosa	0	0	0
Hebdomadis	Hedbomadis	0	0	0
Icterohaemorrhagiae	Icterohaemorrhagiae	0	0	0
Javanica	Poi	0	0	0
Louisiana	Louisiana	0	0	0
Mini	Mini	0	0	0
Pomona	Pomona	0	0	0
Pomona	Altodouro	0	0	0
Pyrogenes	Pyrogenes	0	0	0
Sejroe	Hardjo	0	0	0
Semaranga	Patoc	0	0	0
Tarrassovi	Tarrassovi	1:30	1:30	0

Each GWTS isolate was tested for agglutination by the microscopic agglutination test (MAT) against a panel of reference antisera representative of 19 serovars and 17 serogroups of leptospires. Titres are as indicated. No significant reactivity was detected.

**Table 2:** MAT titres of reference serogroup antigens with antisera specific for each GWTS Isolates 1, 2 & 3.

Reference antigens		Antisera		
Serogroup	serovar	α-GWTS-1	α-GWTS-2	α-GWTS-3
Andamana	Andamana	1:10	0	0
Bataviae	Bataviae	0	1:10	0
Hebdomadis	Kremastos	0	0	0
Hursbridge	Hursbridge	0	0	0
Lyme	Lyme	0	0	0
Louisiana	Louisiana	0	0	0
Louisiana	Orleans	0	0	0
Ranarum	Pingchang	0	0	0
Sarmin	Cuica	0	0	0
Sarmin	Weaveri	0	0	0
Shermani	Aquaruna	1:100	1:30	0
Shermani	Shermani	1:1000	1:3000	0
Undesignated	Sichuan	0	0	0

Antisera specific for each GWTS isolate was tested by the microscopic agglutination test (MAT) against a panel of reference strains of *Leptospira* representative of 9 serogroups and 11 serovars. Titres are as indicated.

### Molecular classification of GWTS isolates of leptospires

**Fig 1:**
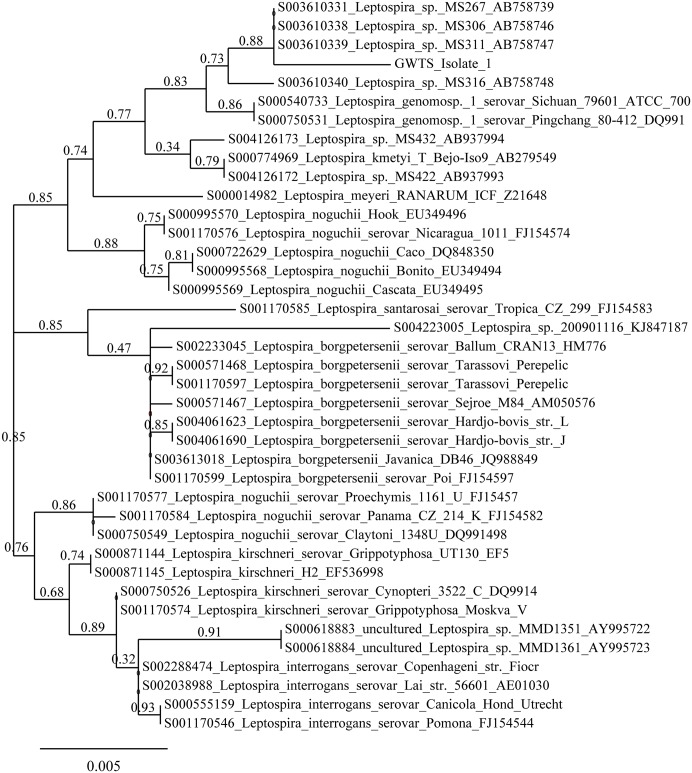
Phylogeny based on 16S rDNA. Phylogenetic reconstruction based on maximum likelihood estimation. Branch lengths are proportional to the number of substitutions per site and branch values are the bootstrap values assigned to the edges (i.e. the branch support values).

**Fig 2:**
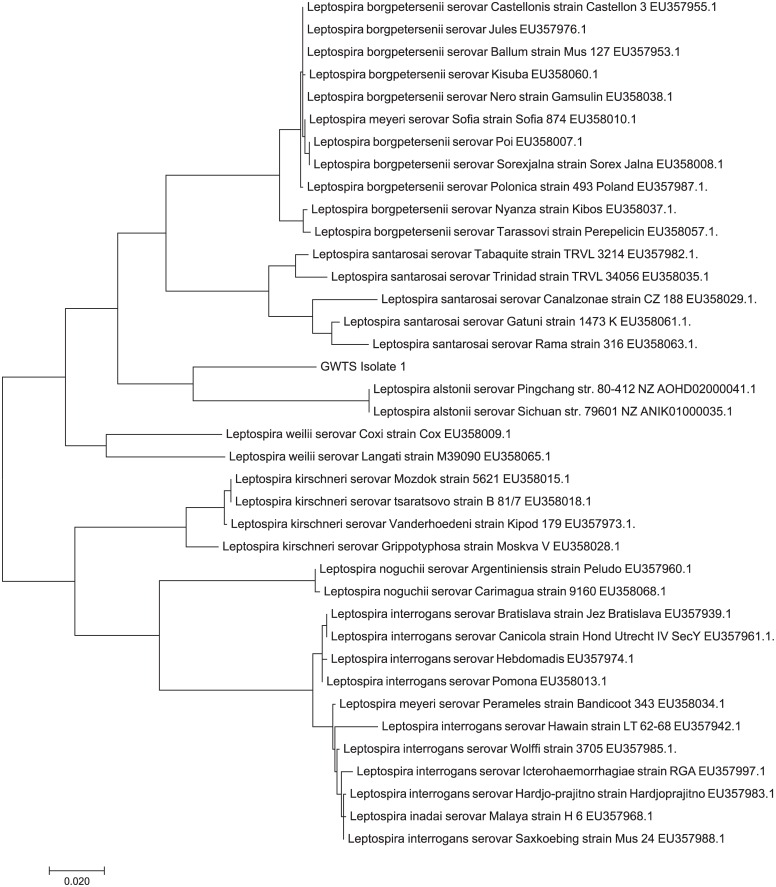
Phylogeny based on *secY*. Phylogenetic reconstruction was inferred using the maximum likelihood method. The tree is drawn to scale, with branch lengths measured in the number of substitutions per site.

The inability to serologically type the GWTS *Leptospira* isolates using reference antisera and reference antigens indicates that the GWTS *Leptospira* isolates are atypical compared to those previously identified in Western Europe. Therefore, whole genome sequencing was performed on a single strain, GWTS isolate #1. The gene sequence for 16S rDNA was extracted from the complete genome and compared to 108 16S rDNA sequences available for *Leptospira* from the Ribosomal Database project (https://rdp.cme.msu.edu/). Phylogenetic analysis indicated that GWTS isolate #1 clustered among 4 strains of *Leptospira* recently isolated from soil samples in Fukuoka, Japan (designated as MS267, MS306, MS311, and MS316 respectively[48]), Fig 1 and S1 Fig. These, in turn, cluster most closely with *Leptospira* genomospecies 1, which has recently been renamed *L*. *alstonii*, and is comprised of two serovars of *Leptospira* that were originally isolated from frogs in China[49]; serogroup Ranarum serovar Pingchang and serogroup Undesignated serovar Sichuan. Similarly, the sequence for *secY* was extracted from the genome and phylogenetic analysis performed; the *secY* sequence of GWTS isolate #1 aligned most closely with that of *L*. *alstonii* serovar Pingchang and *L*. *alstonii* serovar Sichuan, Fig 2. However, rabbit antiserum specific for GWTS isolate #1, 2 or 3, failed to agglutinate with either of these two serovars representative of *L*. *alstonii*, Table 2. Nucleotide sequence for 16S rDNA and *secY* of GWTS #1 is provided (S2 Fig).

**Fig 3:**
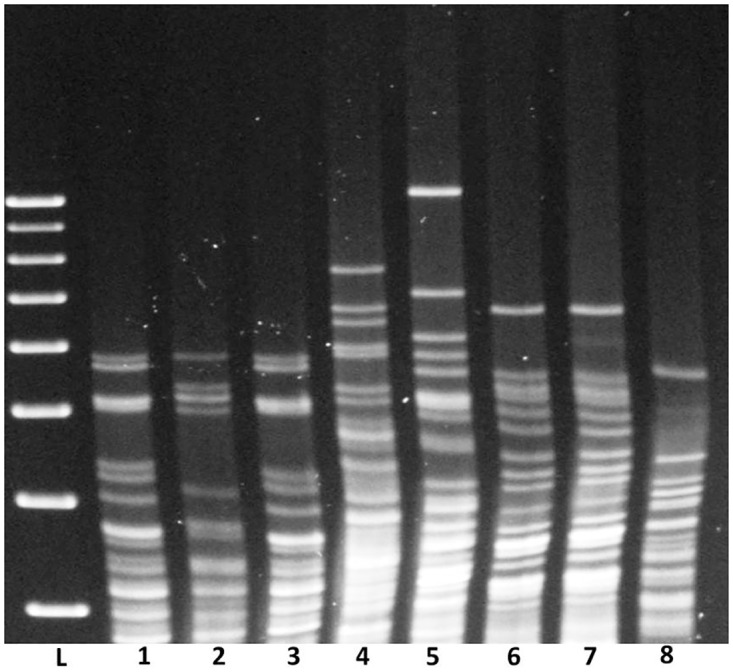
Restriction Enzyme Analysis of GWTS isolates of *Leptospira*. Genomic DNA from GWTS isolates #1 (1), #2 (2) and #3 (3) were compared by REA to that of *L*. *alstonii* isolates of serovar Pingchang (4), serovar Sichuan (5), MS 267 (6), MS 311 (7) and MS 316 (8). L = DNA Marker.

Restriction enzyme analysis was performed on DNA purified from each GWTS isolate #1, 2 & 3 for comparison with 5 of the 6 available isolates of *L*. *alstonii* that have been cultured to date, Fig 3. Results indicate that GWTS isolate #1 and #3 have an identical REA pattern that differed slightly from that of GWTS isolate #2. Results also indicate that the REA patterns are significantly different to that of any of the *L*. *alstonii* isolates. Analysis of REA patterns compared with a reference database of *Leptospira* strains held in the OIE Reference Laboratory (AFBI Stormont, Northern Ireland) did not identify any similar REA patterns.

Collectively, these results provide evidence of the unique and novel molecular attributes of each of the GWTS isolates, which we designate as *L*. *alstonii* serogroup Undesignated serovar Room22.

### Pathogenicity of GWTS Isolates

*Leptospira alstonii* is considered to be a member of the pathogenic complex of *Leptospira*, as defined by DNA-DNA relatedness, 16S rDNA and *secY* sequence. In addition to these criteria, the genome sequence of GWTS#1 contains *lipL32*, which to date has only been identified in pathogenic leptospires (S2 Fig). Each of the GWTS isolates was also tested for growth in the presence of 8-azagunaine; as with all pathogenic leptospires, none of the shrew isolates were able to grow in the presence of 8-azaguanine.

To further assess virulence properties of GWTS isolates, 3 groups of three hamsters were experimentally inoculated with 10^7^ leptospires of GWTS isolate #1, #2 and #3 respectively. No hamster showed any sign of acute disease as determined by weight gain which remained comparable to non-infected controls at all times. All experimentally infected hamsters seroconverted, Table 3, as determined by a positive MAT titre on sera collected at 3 weeks post-inoculation. Sera from experimentally infected hamsters were only reactive with the challenge isolate; no cross-reacting MAT titres were detected when tested against an MAT panel representative for Ireland, and which included serogroup Bratislava, Canicola, Grippotyphosa, Hardjo, Icterohaemorrhagiae or Pomona. Kidneys from experimentally infected hamsters were culture negative for leptospires.

**Table 3:** MAT results of hamsters infected with GWTS isolates.

Challenge isolate and Animal number		GWTS #1	GWTS #2	GWTS #3	B	Ca	G	H	Co	P
GWTS #1	1	1:800	1:800	1:800	neg	neg	neg	neg	neg	neg
	2	1:400	1:800	1:400	neg	neg	neg	neg	neg	neg
	3	1:800	1:1600	1:800	neg	neg	neg	neg	neg	neg
GWTS #2	4	1:1600	1:1600	1:800	neg	neg	neg	neg	neg	neg
	5	1:800	1:800	1:400	neg	neg	neg	neg	neg	neg
	6	1:800	1:400	1:400	neg	neg	neg	neg	neg	neg
GWTS #3	7	1:800	1:800	1:1600	neg	neg	neg	neg	neg	neg
	8	1:800	1:800	1:800	neg	neg	neg	neg	neg	neg
	9	1:800	1:800	1:800	neg	neg	neg	neg	neg	neg

Antisera from hamsters infected with GWTS isolate #1 (animal numbers 1, 2 & 3), GWTS isolate #2 (animal numbers 4, 5 & 6) or GWTS isolate #3 (animal numbers 7, 8 & 9) was tested against each challenge isolate or against a standard MAT panel as indicated; B = serovar Bratislava, Ca = serovar Canicola, G = serovar Grippotyphosa, H = serovar Hardjo, Co = serovar Copenhageni and P = serovar Pomona. Sera from negative control hamsters did not react with any antigen. neg = not reactive.

### Fluorescent antibody test

**Fig 4:**
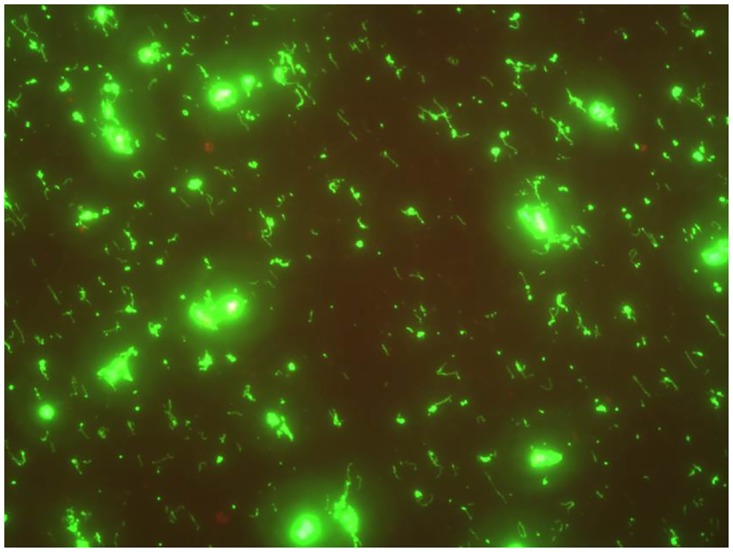
Fluorescent antibody test of GWTS #1. GWTS isolate #1 is reactive with fluorescent conjugated antibody as routinely used to detect leptospires in infected animal tissues.

Serological evidence indicates that each of the GWTS isolates have uncharacterized antigens that fail to mediate agglutination, the basis of current standard typing and diagnostic methodologies. Since FAT is routinely used on infected host tissue to detect leptospires *in situ* by specialist laboratories, an FAT test was performed to determine reactivity with GWTS isolate #1, Fig 4. The positive result indicates that antibody prepared for the detection of leptospires by FAT is able to detect conserved antigens expressed by GWTS isolates.

## Discussion

This study demonstrates that an invasive mammalian species identified in Ireland is infected with a novel bacterial pathogen, designated *L*. *alstonii* serogroup Undesignated serovar Room22. This pathogen has not previously been identified in Ireland, or Europe, and never before been cultured from a mammalian host. Whilst there have been numerous accidental or deliberate introductions of mammalian and avian species into Europe[50], the GWTS population established in Ireland is most likely sourced from within Europe[3]. Regardless, invasive species have unique attributes to facilitate the dissemination of emerging infectious diseases[51]: firstly, invasive species may be more efficient at transmitting pathogens and, as in the case of our study, novel and as yet undescribed, pathogens. Secondly, invasive species tend to thrive in heavily anthropogenic habitats thus increasing the risk of transmission to humans. Thirdly, invasive species tend to have high dispersal rates as exemplified by the GWTS in Ireland with estimates of radial expansion rates of 5.5 km/yr[2]. Finally, invasive species facilitate the establishment of new emerging infectious diseases which are potentially zoonotic.

Leptospirosis is one of the most geographically widespread zoonotic diseases in the world[52]. Historically, all pathogenic leptospires were classified as *Leptospira interrogans* (sensu lato) which were subdivided into serovars, a division based on shared agglutinating lipopolysaccharide antigens and for which more than 200 serovars have been described[53, 54]. With the advent of genomics, pathogenic species of leptospires are now divided into 10 species, based on *in silico* hybridization of whole genome sequences, and include *Leptospira alexanderi*, *L*. *alstonii*, *L*. *borgpetersenii*, *L*. *interrogans* (sensu stricto), *L*. *kirschneri*, *L*. *kmetyi*, *L*. *mayottensis*, *L*. *noguchii*, *L*. *santarosai* and *L*. *weilii* [55–57]. However, the serologic and genomic based typing mechanisms are not mutually exclusive, as exemplified by serovar Hardjo, a significant pathogen in bovine populations throughout the world[58], which may belong to either *L*. *interrogans* or *L*. *borgpetersenii*. Nevertheless, the serologic classification of leptospires continues to play an important role in the epidemiology of leptospirosis and is the basis for the current “gold standard” serologic diagnostic assay, the microscopic agglutination test (MAT). In the MAT, serum from a patient (human or animal) is incubated with a panel of serovars of leptospires to test for a positive agglutination reaction, with the selected panel being representative of a geographical region; one of the obvious limitations of this assay is the composition of the diagnostic panel which will remain negative if tested with serum from a patient that is infected with a serovar not represented in the panel. Such is the case in our studies; when *L*. *alstonii* serovar Room22 was used to inoculate hamsters, all hamsters seroconverted and were MAT positive when tested against serovar Room22; but all were negative, with no cross-reactivity, when tested against six common pathogenic serovars, as typically found in Ireland. Nor was specific antiserum for *L*. *alstonii* serovar Room22 reactive with a range of pathogenic leptospires (Tables 1 and 2). Thus, prior to this study, no mammalian isolate of *L*. *alstonii* was ever available for serological diagnostics by MAT.

*L*. *alstonii* has been cultured from a mammalian host for the first time. Prior isolates of *L*. *alstonii* are derived from the amphibians *Bombina orientalis* and *Rana nigromaculata*, which belong to *Neobatrachia* species in China, or are derived from soil samples in Japan or Malaysia [48, 55, 59]. Whether *L*. *alstonii* serovar Room22 is pathogenic for domestic or wild animal species in Ireland or other parts of Europe and Northern Africa in which the GWTS exists, remains to be determined; such studies can now be facilitated, either by a comprehensive seroprevalence study by MAT, or culture, from other animal species. Alternatively, specialist *Leptospira* laboratories use fluorescent antibody testing (FAT) to detect leptospires in host infected tissue using polyclonal antibodies which cross reacts with *L*. *alstonii* serovar Room22 (Fig 4).

Our results suggest that the GWTS acts as a reservoir host for *L*. *alstonii*. Three isolates of *Leptospira* were identified, none of which had could be typed according to standard serological typing assays for *Leptospira*. Genome sequencing identified GWTS#1 as belonging to *L*. *alstonii*; restriction enzyme analysis (REA) confirmed that GWTS#3 has an identical pattern to that of GWTS#1, which differed slightly to that of GWTS#2. All REA patterns were different to that of other strains of *L*. *alstonii* cultured to date (Fig 3). Similarly, GWTS isolates have no agglutinating titres when tested against the reference strains of *L*. *alstonii* or conversely, when antisera specific for each of the GWTS isolates was test against more recently acquired strains of *L*. *alstonii*. In contrast to incidental hosts which typically suffer an acute limited disease that may include symptoms that range from a mild fever to more severe icteric disease with limited urinary excretion, reservoir hosts are asymptomatic, and may be MAT negative despite persistent renal colonization and excretion of leptospires via urine into the environment[60, 61]. Unique associations between specific host species and certain serovars of leptospires have been recognized; for example, *Rattus norvegicus* acts as a reservoir host for serovar Copenhageni and cattle are reservoir hosts for serovar Hardjo. Both serovar Copenhageni and serovar Hardjo can cause lethal infections in non-reservoir hosts. Whilst the GWTS likely acts as a reservoir host for *L*. *alstonii* serovar Room22, no evidence for acute or chronic disease was detected when serovar Room22 was used to experimentally infect hamsters. These results are similar to those previously described for soil isolates of *L*. *alstonii* in Japan and in which the authors concluded that such results likely reflect attenuation of strains due to continued maintenance under *in vitro* laboratory conditions[48]. Alternatively, a more appropriate animal model is required; in any case, culture of *L*. *alstonii* from the kidneys of the multiple GWTS confirms its pathogenicity. More recently, an *in silico* analysis of 102 isolates of *Leptospira* included the genomes of 3 strains of *L*. *alstonii* as originally isolated from amphibians in China[55]; results not only confirm that *L*. *alstonii* is a pathogen, but that the independent lineages of *L*. *alstonii* gained 504 genes (including three virulence genes) during its evolution, whilst no gene loss was observed. Such observations are interpreted to facilitate the adaptation by *Leptospira* to different hosts and an expanding range of environments.

The GWTS was originally identified in Ireland from skeletal remains in the pellets of barn owls (*Tyto alba*) and kestrels (*Falco tinnunculus*). Barn owls are susceptible to leptospirosis[62]. However it remains to be determined if birds of prey in Ireland are also infected with *L*. *alstonii* serovar Room22, or indeed if the decline of the native pygmy shrew in those areas inhabited by the GWTS is due in part to incidental infection with serovar Room22. There is little information available to assess the implications of the GWTS and associated pathogens on domestic animals and wildlife.

Our results raise additional questions yet to be answered; did the GWTS bring serovar Room22 to Ireland or did it acquire it in Ireland? There is no evidence of serovar Room22 in Ireland prior to capture of GWTS, but nor is there evidence of it in Western Europe or in Africa. Does serovar Room22 infect other domestic or other wild animal species? Up until now, this question could not be addressed by conventional serological surveys. The availability of an isolate of *L*. *alstonii* serovar Room22 from the current studies provides for an isolate to be included in conventional MAT panels, and for the preparation of specific antiserum that can be used in immunohistochemistry or FAT. Molecular assays are still applicable e.g. for the detection of *lipL32*, but such assays do not routinely type positive samples and still rely on a cultured isolate. This was the case in two recent surveys of the greater white-toothed shrew in Germany[63, 64]; in one study, 5 of 24 kidneys were PCR positive for *lipL32*[64]. Additional molecular typing suggested that kidneys were positive for *L*. *kirschneri* but results are not conclusive since the serovar was not identified. Culture was not attempted in either study.

The findings of the current study highlight the importance of screening wildlife for diseases. The current focus on wildlife health surveillance is primarily on human and livestock diseases that are outside the domestic and domiciled environments[65]. This emphasizes a lack of appreciation for the role that sylvatic ecosystems have in the development of zoonotic diseases[28, 66]. To carry out effective wildlife surveillance of emerging infectious diseases that are zoonotic or otherwise, there is a requirement to apply a systematic collaborative approach with veterinarians, ecologists, medical doctors, wildlife biologists, microbiologists and molecular biologists[67]. To date the surveillance of emerging diseases in wildlife is inherently passive[67]. There are clear conservation biology implications of this finding in conjunction with domestic animal health, and potentially human health. Globalization means there are likely to be more introductions of invasive species and therefore societies need to be in position to respond to the effect that these species and their associated pathogens and parasites have on ecosystems[51]. The current study demonstrates precisely what unwanted gifts an invasive species can bear but, to date, the exact consequences of such gifts have yet to be determined.

## Supporting Information

S1 Fig Phylogeny based on 16S rDNA. Phylogenetic reconstruction based on maximum likelihood estimation. Branch lengths are proportional to the number of substitutions per site and branch values are the bootstrap values assigned to the edges (i.e. the branch support values).
(PDF)

S2 Fig Gene sequences for 16S rDNA, *secY* and *lipL32*, as extracted from the whole genome sequence of GWTS Isolate #1. (DOCX)
