# Supplementary figures and images for: Emerging Infectious Disease Implications of Invasive Mammalian Species: The Greater White-Toothed Shrew (Crocidura russula) Is Associated With a Novel Serovar of Pathogenic Leptospira in Ireland
Source: PLoS Negl Trop Dis. 2016 Dec 9;10(12):e0005174. doi: 10.1371/journal.pntd.0005174 (PMC5147805; doi:10.1371/journal.pntd.0005174)

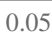

Supplement: S1 Fig — Phylogenetic reconstruction based on maximum likelihood estimation. Branch lengths are proportional to the number of substitutions per site and branch values are the bootstrap values assigned to the edges (i.e. the branch support values). (PDF) [file pntd.0005174.s001.pdf]
