# Supplementary material for: Emerging Infectious Disease Implications of Invasive Mammalian Species: The Greater White-Toothed Shrew (Crocidura russula) Is Associated With a Novel Serovar of Pathogenic Leptospira in Ireland
Source: PLoS Negl Trop Dis. 2016 Dec 9;10(12):e0005174. doi: 10.1371/journal.pntd.0005174 (PMC5147805; doi:10.1371/journal.pntd.0005174)
Supplement: S2 Fig — (DOCX) [file pntd.0005174.s002.docx]

**Supplementary Figure 1**

Gene sequences for 16S rDNA, *secY* and *lipL32*, as extracted from the whole genome sequence of GWTS Isolate #1 are provided as follows:

>GWTS 16S ribosomal DNA

agagtttgatcctggctcagaactaacgctggcggcgcgtcttaaacatgcaagtcaagc

ggagtagcaatactcagcggcgaacgggtgagtaacacgtgggtaatcttcctccgagtc

tgggataacttttcgaaagggaagctaatactggatagtcccgagaggccacaaggcttt

tcgggtaaagattcattgctcggagatgagcccgcgtccgattagctagttggtgaggta

atggctcaccaaggcgacgatcggtagccggcctgagagggtgttcggccacaatggaac

tgagacacggtccatactcctacgggaggcagcagttaagaatcttgctcaatgggggga

accctgaagcagcgacgccgcgtgaacgatgaaggtcttcggattgtaaagttcaataag

cagggaaaaataagcagcaatgtgatgatggtacctgcctaaagcaccggctaactacgt

gccagcagccgcggtaatacgtatggtgcaagcgttgttcggaatcattgggcgtaaagg

gtgcgtaggcggacatataagtcagatgtgaaaactgggggctcaactctcagcctgcat

ttgaaactatatgtctggagtttgggagaggcaagtggaattccaggtgtagcggtgaaa

tgcgtagatatctggaggaacaccagtggcgaaggcgacttgctggcctaaaactgacgc

tgaggcacgaaagcgtgggtagtgaacgggattagataccccggtaatccacgccctaaa

cgttgtctaccagttgttgggggttttaaccctcagtaacgaacctaacggattaagtag

accgcctggggactatgctcgcaagagtgaaactcaaaggaattgacgggggtccgcaca

agcggtggagcatgtggtttaattcgatgatacgcgaaaaacctcacctaggcttgacat

ggagtggaatcatgtagagatacatgagccttcgggccgcttcacaggtgctgcatggtt

gtcgtcagctcgtgtcgtgagatgttgggttaagtcccgcaacgagcgcaaccctcacct

tatgttgccagcattcagttgggcactcgtaaggaactgccggtgacaaaccggaggaag

gcggggatgacgtcaaatcctcatggcctttatgtctagggcaacacacgtgctacaatg

gccggtacaaagggtagccaactcgcgagggggagctaatctcaaaaagccggtcccagt

tcggattggagtctgcaactcgactccatgaagtcggaatcgctagtaatcgcggatcag

catgccgcggtgaatacgttcccggaccttgtacacaccgcccgtcacaccacctgagtg

gggagcacccgaagtggtctttgccaaccgtaaggaagcagactactaaggtgaaactcg

taaagggggtgaagtcgtaacaaggtagccgtatcggaaggtgcggctggatcacct

>GWTS *secY* atgctgaacacttttaaaaacatatttagaattccggagttacgccagaaaattattttt

actctgagcatgcttctgttgttccgtatgggtacacacattacgattcccggcgtcaac

cctgtggttgttgcgggaatcgcaaacgatccatcttccgaaggacttctcggaatggtg

gatcttttcgcgggtggagctttgttaaaattctccatcttcgcactcgggatcatgcct

tacatttcttcatcgatcgtaatgcagttgttcatggtgctcgttccttctcttcaaaaa

cttcaaaaagaaggagaagaaggaagaaagaaaatcggccagtacactaaatacggaacc

gtaatcctttgtgcgattcaatctttagccgtgattcaactcgcaaaaggttggtctacg

ggaaccgaactcgagcccgcaagatatcccggactgatcaactctggcgttattccttat

ttctatttaatcggaatcttatccattaccaccggaaccgttcttttgatctggctcggt

gaacaaattacggaaagaggaatcggaaacggtatttctcttttgatctttgctggtatt

atcggaagacttcctgaatctatggttcaacttttttccaccgatactatggacgctctg

aatgtactgattcttttgattctttttattcttctcatttctcttaccgttttgttaaca

caaggtgtgagaaaagttcctcttcaatacgggaaacagatggtaggaagaaagatggtt

caggcgaaaagccagtccattcctttcaaagtaaacggcgcgaacgtaatgccgattatc

tttgcttcttctttgatcttgtttccacagacgatcattcagtggttgtcgtcgagtagc

gaacagtgggcgggttgggcgatcattatggactttttcaatccattctcccagatctgg

tatcacgcattattctattttatcatctatacttctttgatcgtattcttcgcatatttt

tatactgcgattcagttcaacccgaccgagttggctgagaacttgaagaaatacggcggg

ttcattccagggattcgtccgggttctcatacaaaagaatatatcgaaaaagtgttaaac

agaattactcttccgggcgcgatgttcctggcaggtctggctctggctccttatatcatc

atcaaattcttagatttgagctccaattcgggcggcggatctttggtttatactttcggt

ggaacttctcttttgatcatggtaggggttgcactggagactttgaaacaaatcgagtct

caacttttaatgagaaattatgaaggcttcatgaagaagtcgaaaattaagggaaggtct

taa

>GWTS *lipL32*

atgaaaaaactttcgattttggctatctccgtcgcactctttgcaagcatcacagcttgt

ggagcattcggcggtctgccaagcctaaaaagctcttttgtactgagcgagagcaccatc

ccagggacaaatgaaacagtaaaaactttgctgccctacgggtctgtaatccattactat

ggatacatcaagccaggacaagcgccagacggtttagtcgatggaaacaaaaaagcatac

tacctctacgtttgggttcctgctgttatcgctgagatgggagttcgtatgatttcccct

acaggcgaaatcggtgagccaggcgacggagatctagtaagtgacgctttcaaagctgca

actccagaagaaaaatcaatgccaagttggtttgatacctggattcgcgttgaaagaatg

tcggctattatgcctgaccaaatcgctaaagctgcgaaagcaaaagcacttcaaaagctt

gatgacgatgatgatggagatgatacttacaaagaagagagacatgcaaaatataactct

cttacaagaattaccatccctaatcctccaaaatcttttgacgaactgaaaagtatcgat

actaaaaaacttttagtaagaggtctttacagaatttctttcactacctacaaaccaggt

gaagtgaaaggatctttcgttgcatctgttggtctgctcttcccaccaggtattcctggc

gtgagcccactgattcactcaaatcctgaagaactgcaaaaacaagcagtagctgctgaa

gagtctttgaaaaaagctgcagctgacgctactaagtaa
